# Lipidomic Profile and Enzymes Activity in Hepatic Microsomes of Rats in Physiological and Pathological Conditions

**DOI:** 10.3390/ijms23010442

**Published:** 2021-12-31

**Authors:** Tomasz Lepionka, Małgorzata Białek, Marian Czauderna, Michał Szlis, Agnieszka Białek

**Affiliations:** 1 Department of Bioaerosols, The Biological Threats Identification and Countermeasure Center of the General Karol Kaczkowski Military Institute of Hygiene and Epidemiology, Lubelska 4 St, 24-100 Pulawy, Poland; tomasz.lepionka@wihe.pl; 2 Department of Bromatology, Medical University of Warsaw, Banacha 1, 02-097 Warsaw, Poland; 3 Department of Animal Nutrition, The Kielanowski Institute of Animal Physiology and Nutrition, Polish Academy of Sciences, Instytucka 3, 05-110 Jablonna, Poland; m.bialek@ifzz.pl (M.B.); mr.czauderna@gmail.com (M.C.); m.szlis@ifzz.pl (M.S.); 4 Department of Biotechnology and Nutrigenomics, Institute of Animal Genetics and Biotechnology of Polish Academy of Sciences, Postępu 36A Jastrzębiec, 05-552 Magdalenka, Poland

**Keywords:** pomegranate seed oil, bitter melon extract, dietary supplements, fatty acids, COX-2, cytochrome P450, hepatic microsomes, SPRD rats

## Abstract

Among the risk factors affecting the development of cancer, nutritional factors occupy a significant place. Pomegranate seed oil (PSO) and bitter melon extract (BME), used for ages in folk medicine, are nowadays used in the prevention of many diseases and as ingredients of dietary supplements. Despite numerous publications on these raw materials or their active substances, their mechanism of action in various pathological states has not been recognized yet, nor has the safety of their simultaneous use been evaluated. The study aimed to assess how dietary supplementation with either PSO, with BME, or both, affects fatty acids’ profiles and their metabolism in hepatic microsomes, as well as the activity of selected microsomal enzymes (COX-2 and CYP1B1). Experimental animals (Sprague-Dawley rats) were divided into eight parallel experimental groups, differing in applied dietary modifications (control, PSO, BME and both PSO and BME) and introduction of chemical carcinogen—7,12-dimethylbenz[a]nthracene. Obtained results indicated the pronounced effect of the cancerous process on lipid metabolism and demonstrated the antagonistic effect of applied dietary supplements on the content of individual fatty acids and the activity of CYP1B1 and COX-2. The applied broad analytical approach and chemometric data analysis confirmed that raw materials, for which potential cancer prevention has been previously demonstrated, may differ in effects depending on the coexisting pathological state.

## 1. Introduction

Nutritional factors are considered as the modifiable environmental risk factors of different non-communicable diseases, including cancer. Special attention is lately given to different botanicals, both as herbal medicines and dietary supplements of plant origin, which gain more attention as sources of different bioactive compounds [1]. Conjugated linolenic acids (CLnA) are a group of fatty acids (FA) naturally present in seeds of different plants, among which pomegranate (*Punica granatum*, Lythraceae) and bitter melon (*Momordica charantia*, Cucurbitaceae) are edible plants. CLnA isomers, especially punicic acid (*cis*-9, *trans*-11, *cis*-13 C18:3, PA) found in pomegranate, and α-eleostearic acid (*cis*-9, *trans*-11, *trans*-13 C18:3, αESA) present in bitter melon, are considered as bioactive fatty acids. Different health-promoting activities of CLnA isomers, including their anticarcinogenic properties, are extensively studied. Pomegranate and bitter melon have been widely used both as foods as well as in traditional medicine since ancient times. They are rich sources of polyphenols, fatty acids, vitamins and minerals (pomegranate) or cucurbitane type triterpenoids and glycosides, phenolic acids, flavonoids, carotenoids, essential oils, fatty acids, sterols, saponins, amino acids, some proteins as well as micro- and macroelements and vitamins (bitter melon), respectively [2,3,4]. Because of such a complex composition of diversified bioactive compounds, pomegranate and bitter melon can exert pleiotropic effects, of different mechanisms, and hence, their activity can differ in physiological and pathological conditions or when given simultaneously.

The liver is a multifunctional organ responsible for macronutrients metabolism (including lipids) as well as for detoxification of the organism [5]. Numerous enzymes responsible for these processes are located in hepatocytes, mainly in endoplasmic reticulum (ER), and are known as microsomal enzymes. Hepatic microsomes are heterogeneous vesicles of 20–200 nm diameter, reddish-brown in color, arising from ER when hepatocytes are broken in the laboratory and tissue homogenate is subjected to differential centrifugation. During that process they are concentrated and separated from other cellular debris and subsequently can serve as an in vitro model for enzymes activity studies. There are several microsomal enzymes, including membrane phase I enzymes (cytochrome P450 enzymes (CYPs), flavine-containing monooxygenases (FMOs), esterases, amidases or epoxide hydrolases) and the membrane phase II enzymes, such as glucoronosyl transferases (UGTs) or glutathione-S-transferases, etc. Different factors, such as nutrients, drugs or xenobiotics, can influence the activity of microsomal enzymes directly (by competing for binding to their receptors) or indirectly (by the influence on their gene expression or by changing the microenvironment due to the impact on membrane lipid profile) [6].

It was previously observed that pomegranate seed oil (PSO) and bitter melon fruit extract (BME) can change the cellular membrane lipid profile of different tissues (hepatic, cardiac, mammary) and that this impact depends on the physiological condition or developing pathological condition [7,8,9]. Based on those results we assumed that similar influence can be exerted on an ER lipid profile, which in turn can change the activity of microsomal enzymes. This may be considered as the novel approach to detailed explanation of the mechanisms of PSO and BME action. Moreover, it may indicate the differences in their effects in physiological and pathological conditions. It was previously established that diet modification can influence the lipid composition of microsomal membranes [10]. Moreover, structural changes in microsomal membranes, caused by changes in lipid composition or by incorporation of cycloprenoid fatty acids in the membrane, appeared to be responsible for the lower activities of some microsomal enzymes [11,12]. On the other hand, PSO and BME can directly modify the activity of microsomal enzymes and due to this fact, they can influence both lipid and xenobiotics (e.g., 7,12-dimethylbenz[a]anthracene—DMBA) metabolism. Therefore, the aim of the present study was to examine the influence of PSO and BME in physiological and pathological conditions on the lipid profile and activity of selected enzymes, taking part in fatty acids and xenobiotics metabolism (cyclooxygenase 2—COX2 and isoform 1B1 of cytochrome P450—Cyp1B1) in hepatic microsomes of rats.

## 2. Results

### 2.1. FA Profile in Hepatic Microsomes

Twenty-nine FAs were determined by GC-MS technique in hepatic microsomes. Among them, 9 saturated fatty acids (SFA), 10 monounsaturated fatty acids (MUFA) and 10 polyunsaturated fatty acids (PUFA) were determined (Table 1). SFA and PUFA were present in similar amounts, greatly exceeding the content of MUFA. No significant differences were observed for 8 out of 29 FAs, whereas the other 21 FAs differed significantly among experimental groups. In the Mplus group, SFAs were quantified in the highest amount but significant differences concerned only Mplus and G groups. Similar dependence was obtained for C18:0, which predominated among SFA. C16:0 was present in hepatic microsomes in slightly smaller amounts than C18:0 but its content did not differ among experimental groups. Dietary supplementation with PSO and BME combined with exposure to DMBA resulted in a significant increase in C17:0 content in the GMplus group in relation to CON, M and especially the G group. Similar dependencies were observed in the cases of C15:0 and C20:0, where the microsome content of the GMplus group were the highest. The content of C14:0 tended to be higher in groups of animals exposed to DMBA, especially in the PSO-supplemented Gplus group in relation to groups of healthy animals.

The highest content of MUFA was determined in the Gplus group, which significantly exceeds the total MUFA pool in PSO-supplemented group in physiological state. No differences in c9C18:1 content, which predominated among MUFAs, were determined among experimental groups. Similarly, amounts of t9C18:1, c5C24:1 and c14C18:1, which were detected only in hepatic microsomes of rats treated with DMBA, did not differ among experimental groups. In the cases of both C16:1 isomers, c7C16:1 and c9C16:1, their content in DMBA-treated animals tend to be higher than in healthy animals, with the highest levels quantified in the Gplus group. Similar tendency was observed for c9C17:1, where the highest content in the Gplus and GMplus groups significantly exceeded its amounts in healthy animals. PSO administration to healthy animals resulted in the lowest levels of c11C18:1 of all examined groups. DMBA application tended to increase c11C18:1 content in hepatic microsomes in relation to non-treated animals, with significantly higher content of c11C18:1 in the Mplus group.

Microsomes of the Mplus group were the most abundant in PUFA, where the content significantly exceeded PUFA content in CON and M groups. Hepatic microsomes were composed mainly of n6 PUFA, whose content was about four times higher than n3 PUFA content. It was reflected also in values of n6/n3 ratio, which ranged from 3.29 ± 0.27 in the GMplus group to 3.82 ± 0.30 in the G group. Arachidonic acid (c5c8c11c14C20:4, AA) and linoleic acid (c9c12C18:2, LA) predominated other PUFAs and their higher contents were quantified in the Mplus group. Similarly, in microsomes obtained from the hepatic tissue of this group, the highest level of c6c9c12C18:3 (γ-linolenic acid, GLA) was detected. In the cases of other n6 PUFAs, c11c14C20:2 and c8c11c14C20:3, their significantly elevated accumulation in microsomal fraction was observed in the hepatic tissues of both PSO-supplemented groups which were subjected to DMBA action (Gplus and GMplus). As far as n3 PUFAs are concerned DMBA treatment significantly decreased c9c12c15C18:3 (α-linolenic acid, ALA) accumulation in hepatic microsomes. Similar dependency was observed for c5c8c11c14c17C20:5 (eicosapentaenoic acid, EPA), whose content in CONplus and Mplus was significantly lower than in animals which were not exposed to DMBA. The lowest amount of c7c10c13c16c19C22:5 (docosapentaenoic acid, DPA) was quantified in CONplus, which was significantly lower than in the G, Gplus and GMplus groups. In the case of c4c7c10c13c16c19C22:6 (docosahexaenoic acid, DHA), its highest content was detected in the Mplus group, which significantly exceeded the content of this FA in most healthy animals (CON, M and G groups).

PA and any other CLnA isomers were not determined in the microsomal fraction of livers of animals from any of the experimental groups, even though the supplemented PSO was a rich source of these conjugated fatty acid (CFA) isomers. The GC-MS technique in applied analytical conditions enabled identification and determination in the analyzed biological material of only one conjugated fatty acid—c9t11C18:2 (the CLA isomer; rumenic acid—RA). RA was determined in all groups obtaining PSO supplementation. Its content in physiological state was higher than in animals suffering from breast cancer. The highest content of RA was quantified in hepatic microsomes of GM group, which significantly exceeded its amounts in DMBA-treated animals.

### 2.2. CFA Profile in Hepatic Microsomes

**Figure 1:**
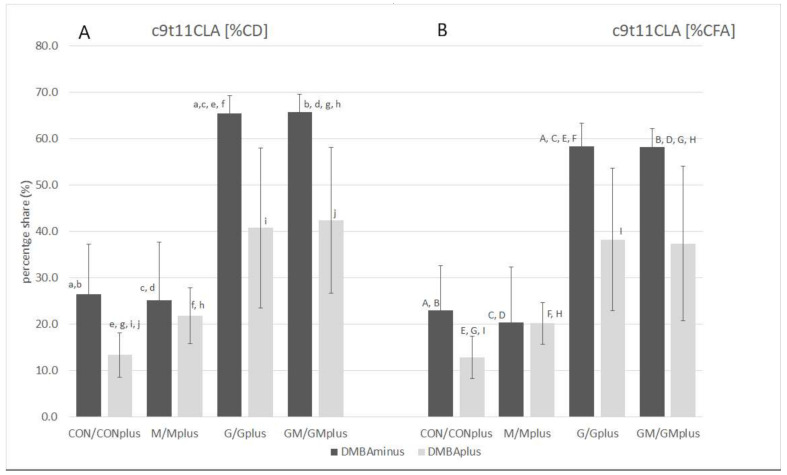
Percentage share of c9t11CLA in CD (**A**) and total CT (**B**) pool in hepatic microsomes of experimental groups. Data are shown as mean values ± standard deviation (SD). Variables marked with common letter indices (a–f and A–F) were significantly different according to Dunn’s test (*p* < 0.05).

**Figure 2:**
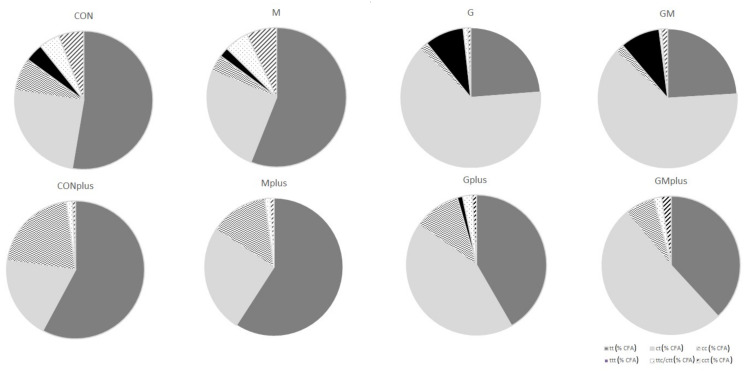
Percentage share of different classes of conjugated fatty acid (CFA) isomers in hepatic microsomes of experimental groups.

Application of Ag^+^-HPLC technique with DAD detection allowed determination of the total profile of CFA isomers incorporated into the microsomal fraction of hepatic tissues of all experimental groups, but their content in PSO-supplemented groups was elevated (Table 2). The highest content of CFA isomers was determined in the GMplus group, which resulted mainly from the high content of conjugated dienes (CD). CDs dominated among CFAs, as their content was several times higher than conjugated trienes (CT). CD share in total CFA ranged from 80.7 ± 23.2% in CON to 97.8 ± 2.0% in Gplus. PSO application resulted in almost ten times higher levels of CD in microsomes compared to groups without PSO supplementation. The highest CD content was determined in the GMplus group, and it was significantly different from the groups not receiving PSO. The proportions of individual CD and CT isomers varied depending on the applied diet supplementation. Among geometrical CD isomers, *trans*, *trans* (tt) CD isomers dominated in the CON and M groups, and CONplus and Mplus groups, while *cis*, *trans* (ct) CD isomers dominated in all groups receiving PSO. Level of tt CD isomers was significantly elevated in PSO-supplemented groups in physiological state and in the GMplus group in pathological state. Similar dependence was observed for ct CD isomers. In the Gplus group the level of tt CD isomers was significantly elevated in comparison with the CON and CONplus groups, whereas ct CD isomers quantity in Gplus dominated over their content in CONplus. RA was the predominant isomer among CD and CFA isomers, which is especially noticeable in groups receiving PSO supplementation (Table 3, Figure 1). Its content in both PSO-supplemented groups in physiological state and in the GMplus group significantly exceeded RA content in other experimental groups. Levels of RA in Gplus were increased to a far lesser extent and they differed only from RA content in the CONplus group. CT isomers constituted 2.2 ± 2.2% in CONplus to 14.9 ± 18.6% in M of total CFA. CT isomers were determined in the highest amounts in the G and GM groups, which exceeded their content in groups not subjected to PSO supplementation. Breast cancer negatively influenced CT isomer incorporation into microsomal fraction of hepatocytes in both PSO-supplemented groups. Among the geometrical CT isomers, *trans*, *trans*, *trans* (ttt) CT isomers were determined in the highest quantity regardless of supplementation or DMBA administration, although their presence was not confirmed in CONplus and Mplus groups. As far as *trans*, *trans*, *cis*/*cis*, *trans*, *trans* ttc/ctt and *cis*, *cis*, *trans* (cct) CT isomers are concerned, their amounts were elevated in the GMplus group in comparison with GM, CONplus and Mplus for ttc/ctt CT isomers and with CON and Mplus for cct CT isomers. Percentage share of individual types of CFA isomers differed regarding PSO supplementation, which resulted in a predominating share of ct isomers in the total CFA pool in PSO-supplemented groups. However, PSO impact depended on physiological or pathological state of the animals, e.g., cc CD isomers share and tt CD isomers share differed significantly (Figure 2).

### 2.3. Chemometric Analysis

**Figure 3:**
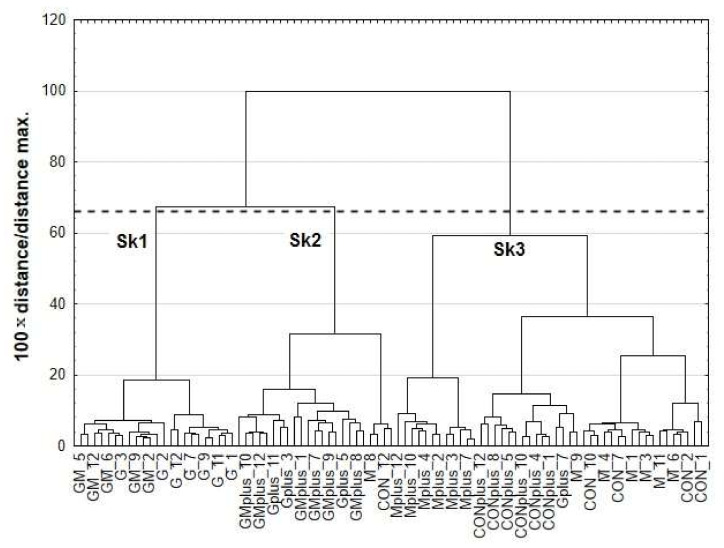
Dendrogram of similarities of FA and CFA profile in the microsomes of experimental groups.

The results of cluster analysis (CA) are presented as dendrograms in Figure 3. The application of Sneath’s criterion (66%) to the dendrogram analysis allowed distinguishing of three clusters (Sk1–Sk3), that group the examined microsomal samples. The first cluster (Sk1) included samples of G and GM groups, whereas Gplus and GMplus samples were incorporated mostly into the second cluster (Sk2). Most samples from CON, M, CONplus and Mplus created the third cluster (Sk3). Sample allocation to distinguished clusters to a large extent coincided with the experimental groups, taking into account both diet and carcinogen treatment. Content of all CFAs and most fatty acids differed significantly among existing clusters. No significant differences were established for cc, cct, C12:0, c7C15:1, C18:0, c6C18;1 c9c12C18:2, c6c9c12C18:3 and c5c8c11c14C20:4 (*p* > 0.05).

**Figure 4:**
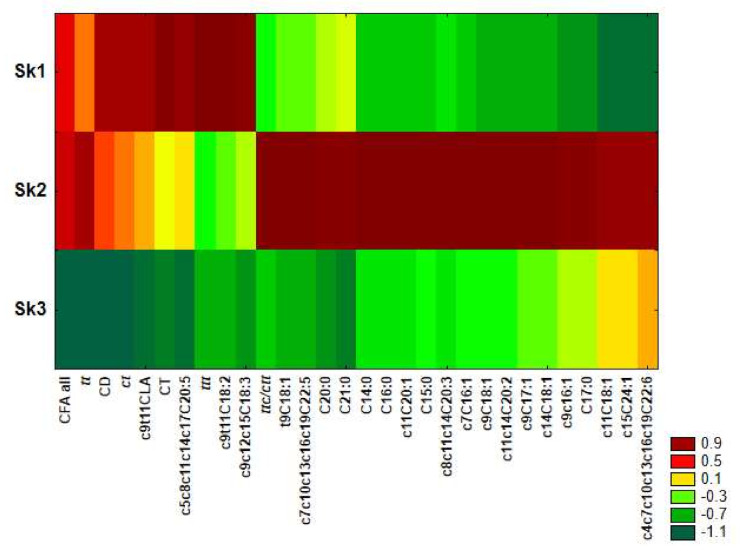
Heat maps of FA and CFA profiles in the microsomes of experimental groups.

Similarity analysis was performed by using the method of grouping features and objects for fatty acids and CFAs differing significantly among clusters revealed in CA. This was applied to prepare a heat map (Figure 4) and clearly shows that serum samples of Sk1 were characterized by the highest share of most of CFAs (apart from ttc/ctt), c9c12c15C18:3, c9c11C18:2 and c5c8c11c14c17C20:5, as well as the lowest share of c11C18:1, c15C24:1 and c4c7c10c13c16c19C22:6. In microsomal samples included in Sk2, the highest amounts of most of the examined fatty acids were detected apart from c9c12c15C18:3, c9c11C18:2 and ttt. For Sk3, low levels of most examined CFAs and fatty acids were distinctive.

Linear discriminant analysis (LDA) was used to obtain appropriate classification rules for examined samples into distinguished clusters (Sk1–Sk3). Relevant discriminant functions were calculated in a stepwise progressive method. In the performed analysis, 17 variables out of all those examined were included in the final model, and 10 of them (c9c11C18:2, c14C18:1, c7C15:1, c15C24:1, ttc/ctt, CT, c6C18:1, ct, c6c9c12C18:3, cc) were significant in the model. Their contribution to overall discrimination was diversified. Applied canonical analysis allowed distinguishing of two statistically significant (*p* < 0.05) discriminant functions (DF). DF1 is the most significant function, as it explains 88.5% of the discriminatory power, whereas DF2 explains 11.5% of discriminatory power (Table 4).

**Figure 5:**
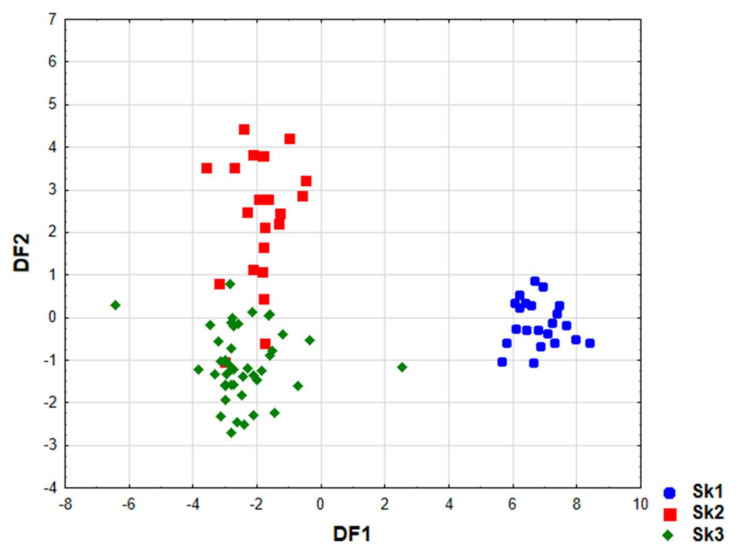
Scatter plot of canonical values for DF1 and DF2 functions.

Analysis of canonical mean variables indicated that DF1 had the greatest impact on the distinction of Sk1 from other clusters and DF2 is responsible for distinguishing Sk2 from Sk3 (Table 4). Graph analysis confirms the suggestion provided by the values of average canonic variables (Figure 5).

The calculated classification matrix indicated that average classification efficiency based on the calculated functions was 94.3% (Table 5). For individual clusters these coefficients were as follows: 100% for Sk1, 97.7% for Sk3 and 81.8% for Sk2, respectively.

### 2.4. Peroxidability Index, LA Isomerization Index and Desaturases Indices

Values of indices, calculated based on FA and CFA content, are shown in Table 6. The highest value of peroxidability index (PI) was determined in the Mplus group, which was significantly higher than two groups of animals not exposed to the chemical carcinogen, CON and M, and for all other groups exposed to DMBA. Moreover, significant difference was also observed between the CONplus and Gplus groups.

Due to the fact that CD isomers were present only in the microsomal fractions of groups receiving PSO, the determination of LA isomerase activity (Iso-LA) was performed only in experimental groups supplemented with this oil. The highest value of Iso-LA was determined in the GM group, which was significantly higher than in the GMplus group.

Values of desaturase indices differed significantly among experimental groups. The highest Δ4-desaturase capacity was found in the CONplus and Mplus groups, for which the Δ4-desaturase index (D4D) values were significantly different from the values of this index determined in the CON, M and GM groups. In the Mplus group the highest value of the Δ5-desaturase index (D5D) was also found, which differed significantly from the results obtained for the other experimental groups, except for the G and CONplus groups. Significantly higher value of the D5D was also found in the G group compared to the Gplus and GMplus groups. Regarding C16 FA, the capacity of Δ9-desaturase was significantly higher in some groups exposed to DMBA: CONplus, Gplus and GMplus in comparison with the CON and GM groups. The value of Δ-9 desaturase index for C18 FA (D9D_C18) was the highest in the Gplus group and it differed significantly from its values obtained for the CON and Mplus groups. As far Δ9-desaturase is concerned, the highest value of the total Δ-9 desaturase index (D9D_total) was found in the Gplus group, which was significantly higher than in the Mplus group.

### 2.5. COX-2 Activity

**Figure 6:**
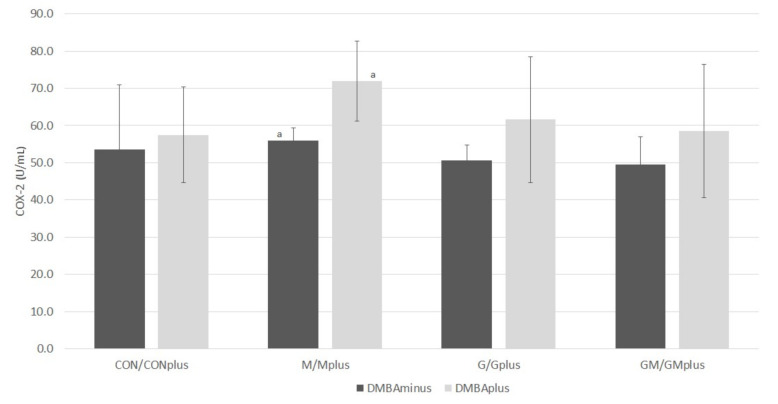
COX-2 activity in hepatic microsomes of experimental groups (U/mL). Data are shown as mean values ± standard deviation (SD). Variables marked with common letter index (a) were significantly different according to Dunn’s test (*p* < 0.05).

Results of COX-2 activity are presented in Figure 6. No differences in COX-2 activity were observed among groups of animals in physiological state. In groups of animals treated with DMBA, the COX-2 activity was similar, however, there was a tendency for it to increase in relation to the groups of healthy animals. The highest activity of this enzyme was observed in the Mplus group, and it was significantly higher than in the M group.

### 2.6. CYP1B1 Content

**Figure 7:**
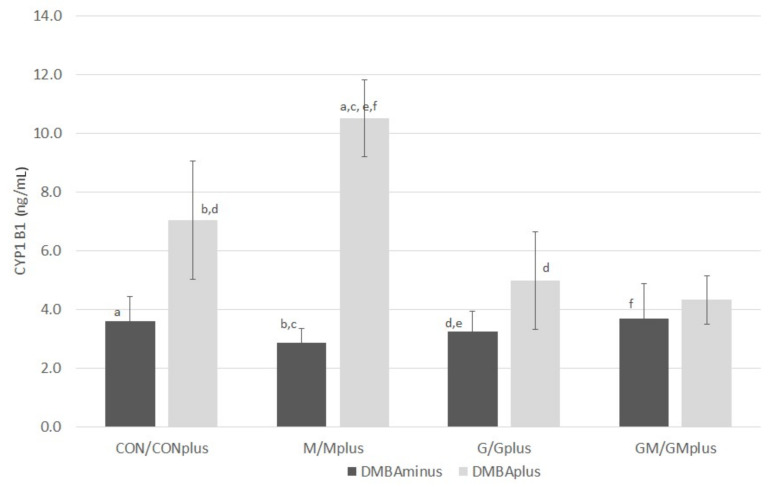
The content of CYP1B1 in hepatic microsomes of experimental groups (ng/mL). Data are shown as mean values ± standard deviation (SD). Variables marked with common letter indices (a–f) were significantly different according to Dunn’s test (*p* < 0.05).

The content of CYP1B1 is presented in Figure 7. In physiological conditions, no significant differences in the levels of this isoform of P450 cytochrome were observed among experimental groups, regardless of the applied dietary supplementation. DMBA treatment tends to increase CYP1B1 amounts in microsomal fraction of hepatocytes. PSO-supplementation tends to decrease the content of this cytochrome isoform in animals suffering from mammary tumors. The highest level of CYP1B1 was quantified in Mplus, which significantly exceeded its content in physiological conditions. Furthermore, its content in CONplus was significantly higher than in the M and G groups.

## 3. Discussion

The microsomal fraction contains different enzymes catalyzing numerous processes, including enzymes responsible for the metabolism of FA, e.g., chain elongation reactions (elongase) or incorporation of double bonds into the chain (desaturases). It was previously established that the cancerous process is characterized by changed metabolism, including increased utilization of lipid compounds, e.g., by uptake from an extracellular matrix or de novo synthesis [13,14,15]. We previously revealed the significant differences in lipidomic profile in serum and tissues (mammary, cardiac, hepatic) generated by developing breast cancer [7,8,9,16]. We hypothesized that modified lipid metabolism in cancerous process may result inter alia from alteration in the activity of enzymes involved in lipid metabolism.

The microsomal fraction contains enzymes related to the endoplasmic reticulum also including various isoforms of cytochrome P450, responsible mainly for catalyzing the phase I reactions of xenobiotic metabolism. Some isoforms of cytochrome P450 also take part in FA and sterols metabolism. Chemical carcinogen DMBA, belonging to polycyclic aromatic hydrocarbons, requires metabolic activation by mixed-function oxidases located in the mammalian cells microsomes to exert its carcinogenic activities. The critical step in this process towards carcinogenic activity in the mammary glands of rats is the hydroxylation of 7-methyl [17]. The activity of cytochrome P450 enzymes depends on the profile of FA in the endoplasmic reticulum. Changes in the proportions of individual FA in diet may result in modifying the properties of cellular and intra-cellular membranes and hence in changes of the activity of signaling pathways, as a result affecting the amount and type of reaction products catalyzed by these enzymes. Moreover, different bioactive fractions obtained from bitter melon and from pomegranate, were confirmed to influence the activity or expression of different isoforms of cytochrome P450 [18,19,20,21,22]. However, no direct influence of dietary supplements originating from bitter melon and pomegranate, which were applied in our experiments, on isoform 1B1 of cytochrome P450 was established. It seems essential to determine the potential impact of the applied dietary supplements and exposure to the procarcinogen—DMBA—on the lipidomic status and activity of enzymes present in the hepatic microsomal fraction.

The results of determination of total FA profile in microsomal fraction indicate that applied supplementation had a negligible effect on changes in the FA profile, especially in the case of healthy animals. In contrast, the impact of carcinogen exposure was more pronounced. Significant changes in the content of individual FAs in supplemented groups compared to the control groups were observed only for a selected few FA (C14:0, cC18:3 n6 GLA in healthy animals and C22:5 acid in animals exposed to DMBA).

The administration of DMBA resulted in significant differences in the content of individual FA in the microsomal fraction between the groups of healthy animals and those exposed to the carcinogen. It also made it possible to demonstrate the antagonistic effect of the applied dietary supplements (PSO and BME) on the content of individual FA. This association can be observed in case of the profile of SFA and MUFA, where the PSO application significantly influenced the content of individual FA compared to the groups receiving BME, which resulted in a significant difference in the total content of saturated FA in microsomes. Similar effect was previously observed in the case of FA profile in serum [16]. However, these results contrast with observations made for whole hepatic tissue [7], which suggests that susceptibility of cellular and intra-cellular membrane lipids on changes induced by dietary factors is diversified.

The applied dietary supplements modulated the lowering effect of DMBA on PUFA levels. PSO-supplementation weakened DMBA’s influence on the content of n3 PUFA: ALA and EPA. Similarly, BME supplementation tended to attenuate the decreasing effect of DMBA on LA levels in the microsomal fraction. Additionally, the use of this supplement significantly increased the content of DHA and AA in individuals exposed to DMBA. Our previous research supports these observations both in liver and serum [7,16].

The determination of the CFA isomer profile in hepatic microsomal fraction was performed with the Ag^+^-HPLC technique with photodiode detection. This technique is highly selective for the determination of structural and geometric CFA isomers, which differ in spectra and the maximum absorption wavelength. Its application seems to be the optimal method research involving dietary supplements with CFA isomers and was previously used in studies on the CLA [23,24] and CLnA profile [9,25]. Elevated content of CFA isomers in the groups supplemented with PSO, which is a rich source of CFA isomers, indicates the successful incorporation of these isomers into the microsomal lipid fraction, preferably as CD—mainly as RA. It is in line with our previous observations, as we detected the presence of PA neither in serum [16] nor in liver [7] or myocardium [9] in any of the experimental groups. These observations are compatible with those made by Kohno et al. who also did not determine PA in the livers of animals supplemented with 0.01, 0.1 and 1% PSO [26]. It was previously shown that PA is completely converted to RA in rat liver tissue, kidneys and intestines and in that form is built into different tissues [27]. In contrast, Yuan et al. identified both PA and αESA in the livers of mice supplemented with 1% of these acids and observed their conversion to RA to a different degree, depending on the type of fatty acid isomer and the type of tissue. In livers, the conversion rate to CLA was 88% for αESA and 76% for PA, respectively [28]. These results explain the significantly higher levels of CD compared to CT determined in the hepatic microsomes of all groups in our experiment. There was a significant increase in the total content of CFA and total contents of CD and CT isomers in all groups supplemented with PSO. However, this effect was modified by the exposure to DMBA, which indicates that the cancerous process may alter the CLnA metabolism. We observed a similar but more pronounced effect regarding PUFA profile in microsomal fraction and our previous experiments in liver tissue and serum [7,16]. A decrease in CFA content due to exposure to DMBA was also observed in our previous research [9,16].

CYP450 enzymes are key enzymes in the hepatic microsomal fraction involved in the metabolism of xenobiotics and the biosynthesis of endogenous compounds, such as lipids and their derivatives. CYP1B1 is involved in the metabolism of sterols, and its increased activity is associated with the occurrence of various types of cancer, including prostate, uterus and breast cancers [29]. We observed an increasing influence of the neoplastic process on the contents of the cytochrome P450 isoform 1B1 in the microsomal fraction. The CYP1 isoenzymes family are a group of cytochromes where expression is regulated by the aryl hydrocarbon receptor (AhR). It is a transcriptive factor activated by specific ligands, such as polycyclic aryl hydrocarbons, e.g., the carcinogen used in this experiment—DMBA. Thus, an observed increase in the activity of CYP1B1 may result from its role in the activation of carcinogenic compounds and the induction of hormone-dependent neoplasms, including breast cancer. Furthermore, the applied dietary supplementation influenced the amount of CYP1B1, although the effect depended on the type of a supplement. The highest content of CYP1B1 in the Mplus group may be due to the activating effect of BME, which results in overexpression of the genes encoding this isoenzyme. The opposite effect of PSO on the expression of CYP1B1 may be due to the influence of significant amounts of PUFA present in this oil on AhR activity, which was previously observed by other authors, who showed diverse effects of dietary PUFA on the expression of CYP1B1 mRNA in the liver and neoplastic tumors of SPRD rats exposed to DMBA [30].

Cyclooxygenases are enzymes that catalyze the synthesis of prostanoids from the substrates, which are free fatty acids, mainly AA, and other PUFA [31]. The expression of the COX-2 gene is stimulated by growth and pro-inflammatory state; therefore, the increase in COX-2 expression is characteristic for degenerative and neoplastic diseases, e.g., in cancers of the colon, stomach, esophagus, breast, lung, endometrium, bladder and prostate [32,33]. The obtained results show that the activity of COX-2 in the microsomal fraction is not susceptible to introduction of PSO. The only significant increase in COX-2 activity was observed in animals exposed to DMBA and supplemented BME. It seems that BME may increase COX-2 activity, while its effect in healthy animals may be opposite. Similar results were obtained by Lii et al., who observed an increase in lipopolysaccharide-stimulated COX-2 gene expression in BME-treated macrophage cells relative to the control group [34]. On the other hand, Chao et al. observed the opposite effect of the powdered *Momordica charantia* fruit, resulting in a reduction in COX-2 expression and the level of its product—prostaglandin E_2_ (PGE2) [35].

Obtained results of PI, calculated based on FA profile, indicates that applied dietary modifications in healthy animals affected microsomal FA profile in terms of susceptibility to peroxidation processes. However, previously we did not observe the effect of supplementation with various doses of CLA and PSO on the PI value in the livers of Sprague-Dawley (SPRD) rats [36]. A significant increase of PI in animals exposed to DMBA and supplemented with BME indicates an increased susceptibility of FA incorporated into ER to oxidation. In contrast, the introduction of PSO seems to have the opposite effect.

Based on FA profile, indices of desaturases activity were also calculated. Desaturases are oxidoreductases associated with the membranes of the endoplasmic reticulum, involved in FA transformation, as they catalyze introducing a double bond during the biosynthesis of the carbon chain. The availability of research regarding the impact of dietary supplements used in our experiment on the activity of desaturases in terms of the neoplastic process is negligible. The observed decrease in D5D activity in the PSO supplemented groups may result from the competition between CLnA (or its metabolites—CLA) with LA for D5D and D6D active sites [37] or from the lowering effect of CFA on AA levels [38]. However, such an effect was not observed in the microsomal fraction. We also observed a significant increase of the D9D activity as an effect of supplementation PSO and the exposure to DMBA, which is surprising regarding observations that high dietary PUFA intake inhibits the expression of genes encoding D9D [39]. It seems that the high value of the D9D index in both PSO supplemented groups results to a greater extent from the coexisting neoplastic process. This assumption is confirmed by Kim et al., who showed a relationship between elevated D9D activity and the occurrence of prostate cancer [40]. Our results indicate that the applied dietary modifications in healthy animals do not cause significant changes in the activity of any of the analyzed desaturases, in contrast to the groups exposed to the carcinogenic factor. Exposure to DMBA resulted in differences in the activity of desaturases not only between groups of healthy and tumor-bearing animals but also within groups exposed to the carcinogenic factor. These observations confirm the influence of the ongoing neoplastic process on the modification of the FA profile, which results in the modified activity of desaturases in the microsomal fraction.

Applied chemometric approach to data analysis confirmed that both co-existing pathological process and PSO administration greatly influences the FA and CFA profile of ER. However, the direction of lipid profile modifications induced by PSO varies in physiological and pathological conditions and in case of many FAs and CFAs it appears to be opposite (Figure 4).

## 4. Materials and Methods

### 4.1. Dietary Supplements: PSO and BME

Cold-pressed, unrefined oil from seeds of pomegranate fruits (pomegranate seed oil, PSO), originating from Great Britain (100%, ECOSPA), was purchased from the local market in Warsaw, Poland. It was protected from light in a dark glass bottle and it was stored unopened at 8 °C in the original manufacturer’s package. It was administered daily to animals via gavage, in the amount of 0.15 mL per animal after bringing it to ambient temperature. Bitter melon tea for brewing (Tra Kho Qua, Hung Phat Corp, Vietnam) consisting of bitter melon dried fruits, was purchased from the local market in Warsaw, Poland. Bitter melon fruits aqueous extract of 1% (*w*/*v*) (BME) was prepared fresh daily. Hot water (80 °C) was added to the weighted portion of the dried material, left for 10 min, filtered and administered to animals after bringing it to ambient temperature. Fresh BME was given to animals as drinking fluid daily ad libitum. Detailed characterization and content of bioactive compounds in both applied botanicals were given previously [16,41].

### 4.2. Animals

The animal experiment was carried out in accordance with European Union Directive 2010/63/EU for animal experiments after obtaining the 2nd Local Ethical Committee on Animal Experiments consent (No. 56/2013 and 54/2015). The detailed design of animal experiment was thoroughly described elsewhere [16]. Sprague–Dawley rats (females, n = 96, age 30 days) were purchased from the Central Laboratory of Experimental Animals, Medical University of Warsaw (Warsaw, Poland). During the entire experiment they were housed in an animal room at a constant temperature of 21 ± 1 °C with 12 h light-dark cycle and relative humidity of 50–60%, in plastic cages (2 individuals per cage). Animals were fed the standard laboratory chow Labofeed H ad libitum (Feed and Concentrates Production Plant, A. Morawski, Żurawia 19, Kcynia, Poland) and had unrestricted access to fresh, drinking fluid (water or BME). The detailed composition of Labofeed H (per kg of diet) was previously published [16]. After a 1-week adaptation period rats were randomly divided into the following eight groups (12 animals per group):

− CON and CONplus—control groups without diet supplementation, fed a standard diet and water *ad libitum*,
− M and Mplus—animals fed a standard diet supplemented with 1% aqueous extract of bitter melon dried fruits (BM) *ad libitum*,
− G and Gplus—animals were fed the standard diet and water *ad libitum* and were given 0.15 mL/d PSO via intragastric gavage,
− GM and GMplus—animals were fed the standard diet and were supplemented with both 0.15 mL/d PSO via intragastric gavage and 1% BM *ad libitum*.

Diet supplementation started after an adaptation period, at 37th day of life and lasted for 21 subsequent weeks. Daily intake of fodder and drinking fluid did not differ among control and experimental groups. For the induction of mammary tumors animals from four experimental groups defined as “plus” were given 7,12-dimethylbenz[a]anthracene (DMBA) at the dose of 80 mg/kg body weight on the 50th day of life. DMBA was administered as a solution intragastrically via gavage after dissolving directly in PSO (in case of Gplus and GMplus groups) or in rapeseed oil (in case of CONplus and Mplus groups). During the whole experiment animals were also monitored on a daily basis for any specific signs of welfare disorders (e.g., appetite loss, ruffling, sluggishness, apathy, hiding, curling up). They were also checked for any specific signs of health deterioration and weighed at weekly intervals. After the experimental period all animals from each group were decapitated and exsanguinated.

### 4.3. Preparation of Hepatic Microsomes

Livers were excised during autopsy and hepatic microsomes were prepared according to the method of Kłyszejko-Stefanowicz with slight modifications, which was thoroughly described previously [42]. Three parallel samples were prepared from liver sample obtained from one animal and they were stored frozen at −80 °C prior to analyses.

### 4.4. Determination of Fatty Acids in Hepatic Microsomes

Fat content in hepatic microsomes was determined according to the procedure of Folch et al. [43]. Thawed samples of hepatic microsome suspension (0.5 mL) were subjected to alkaline hydrolysis according to the method of Czauderna et al. [44] as the first step of sample preparation to chromatographic analyses. Next, fatty acids were derivatized by the base- and acid-catalyzed methylations procedures [45] to obtain FA methyl ester (FAME) and then quantified using a gas chromatograph (Shimadzu GC-MS-QP2010 Plus EI; Tokyo, Japan) equipped with a BPX70 fused silica column (120 m × 0.25 mm i.d. × 0.25 μm film thickness; Phenomenex, Torrance, CA, USA), a quadrupole mass selective detector (Model 5973N) and an injection port [45]. The temperature program of the column was as follows: 70 °C for 6 min, increase to 150 °C at a rate of 12 °C/min and held for 6 min, then increased to 168 °C at a rate of 5 °C/min and held for 27 min, then increased to 190 °C at a rate of 0.75 °C/min and this temperature was maintained for 10 min, then raised to 210 °C at a rate 1.80 °C/min and held for 25 min, then increased to 234 °C at 6 °C/min and held for 4 min, then increased to 236 °C at 6 °C/min and held for 40 min. Helium was used as carrier gas at a flow rate of 1.0 mL/min. Chloroform solution of nonadecanoic acid (C19:0) was used as an internal standard (17 mg/mL). FAME identification was validated based on electron impact ionization spectra of FAME and compared to authentic FAME standards (Sigma, St. Louis, MO, USA) and the NIST 2007 reference mass spectra library (National Institute of Standard and Technology, Gaithersburg, MD, USA). All FAME analyses performed were either based on total ion current chromatograms, selected-ion monitoring chromatograms or both. FA content was expressed in relation to fat content in hepatic microsomes.

### 4.5. Determination of Conjugated Fatty Acid (CFA) Isomers in Hepatic Microsomes

CFA isomers, isolated from hepatic microsomes by saponification and extraction according to [44], were analyzed directly, without precolumn derivatization, using the Ag^+^-HPLC technique. Four analytical ion-exchange columns, loaded with silver ions (Chrompack ChromSpher, 5 μm, Lipids, 250 mm × 4.6 mm; Varian, The Netherlands), connected in series and a Waters HPLC 625LC system equipped with a photodiode array detector (PDA) (Waters, Milford, MA, USA) operating in a UV range from 195 to 400 nm were applied. Ag^+^-HPLC-DAD system pressure was 15.25 ± 0.08 MPa. Mobile phase consisted of n-hexane: acetic acid: acetonitrile (98.5:1.6:0.0125, v/v/v). The flow rate was 2 mL/min and the column temperature was maintained at 23 °C. CFA isomers: conjugated dienes (CD) or conjugated trienes (CT), respectively, were identified based on their retention times and UV spectra of analytical standards (Larodan Fine Chemicals, Solna, Sweden). Sorbic acid (c2,c4C6:2, Sigma, St. Louis, MO, USA) was used as an internal standard [25]. Contents of CFA isomers were expressed in relation to fat content in hepatic microsomes and profiles of CD and CT isomers were expressed in relation to total CFA pool.

### 4.6. Determination of Peroxidability Index, LA Isomerization Index and Indices of Desaturases Activity in Hepatic Microsomes

Based on the fatty acid profile determined in the microsomal fraction, the following desaturase activity indices were calculated: Δ4-desaturase—D4D, Δ5-desaturase—D5D, Δ9-desaturases for C16:0—D9D-C16, Δ9-desaturase in relation to C18:0—D9D-C18 acids, Δ9-desaturases—D9D, as well as the peroxidability index (PI) and the linoleic acid isomerization index—iso-LA, based on the following formulas:

D4D = c4c7c10c13c16c19C22:6/c7c10c13c16c19C22:5

D5D = c5c8c11c14C20:4/c8c11c14C20:3

D9D-C16 = c9C16:1/C16:0

D9D-C18 = c9C18:1/C18:0

D9D = (c9C16:1 + c9C18:1)/(C16:0 + C18:0)

PI = (monoenoic FA × 0.025) + (dienoic FA × 1) + (trienoic FA × 2) + (tetraenoic FA × 3) + (pentaenoic FA × 4) + (hexaenoic FA × 5)

Iso-LA = CD/(c9c12C18:2 + CD),

according to [41,46].

### 4.7. Determination of COX-2 Activity in Hepatic Microsomes

Determination of cyclooxygenase 2 (COX-2) activity in liver microsomes was performed with a commercial ELISA kit (COX Activity Assay Kit, Cayman Chemical Company, type 760151) according to the manufacturer’s instructions.

### 4.8. Determination of CYP2B1 Isoform Activity

Determination of the activity of the cytochrome P-450 isoform 1B1 in liver microsomes was performed with a commercial ELISA kit (Assay Kit for Cytochrome P450 1B1, Cloud-Clone Corp., Houston, TX, USA, type SED297Ra), according to the manufacturer’s instructions.

### 4.9. Statistical Analysis

All data were presented as mean values ± standard deviation. Statistica 13 software was used for the statistical analysis. For variables with normal distribution obtained data were tested with one-way ANOVA and post hoc Tuckey test. For variables without normal distribution data were tested with the Kruskal–Wallis test, which is a non-parametric equivalent of one-way ANOVA, with post hoc Dunn’s test. The acceptable level of significance was established at *p* ≤ 0.05.

In order to verify whether the diet modifications and applied experimental conditions significantly affected group diversity chemometric analyses were performed using Statistica 13 software. FA and CFA content and in hepatic microsomes were used as descriptors to study a possible discrimination of the microsomal samples. Prior to analyses, the original data were transformed into natural logarithms and then auto-scaled (standardized). Cluster analysis was carried out using the agglomeration method. Euclidean distance was used as the distance determination method and the Ward method was used as the agglomeration method. The application of less restrictive Sneath’s criterion (66%) was used for dendrograms analysis and cluster distinguishing. To determine the differences among existing clusters of microsome samples the non-parametric Kruskal–Wallis test with post hoc multiple comparison test was used. The accepted significance level was established at *p* < 0.05. Similarity analysis was performed by grouping of features and objects for variables differing significantly among existing clusters to prepare heat map. Next, in order to obtain appropriate classification rules for microsomes samples into distinguished clusters, a linear discriminant analysis for examined variables (FA and CFA content) differing significantly among clusters was performed. Relevant discriminant functions were calculated in a stepwise progressive method, with the adopted tolerance value 1 – R^2^ = 0.01 to optimize LDA.

## 5. Conclusions

Our results revealed that applied supplementation had a negligible effect on changes in the FA profile, especially in the case of healthy animals. In contrast, the impact of carcinogen exposure was more pronounced. Moreover, the antagonistic effect of the applied PSO and BME on the content of individual FA was observed. In the cases of SFA and MUFA content, PSO application significantly influenced the content of individual FA compared to the groups receiving BME, which resulted in a significant difference in the total content of SFA in microsomes. PSO-supplementation weakened DMBA influence on the content of n3 PUFA: ALA and EPA. Similarly, BME-supplementation tended to attenuate the decreasing effect of DMBA on LA levels in the microsomal fraction. Additionally, the use of this supplement significantly increased the content of DHA and AA in individuals exposed to DMBA. Both diet supplementations and cancerous process also modulated the CFA profile of ER and influenced the activity of microsomal enzymes. An increasing influence of the neoplastic process on the contents of the cytochrome P450 isoform 1B1 in the microsomal fraction as well as the opposite effect of PSO and BME were observed. The activity of COX-2 in the microsomal fraction was not susceptible to introduction of PSO and the only significant increase in COX-2 activity was observed in animals exposed to DMBA and supplemented with BME, which suggests that BME may increase COX-2 activity in pathological conditions while its effect in healthy animals may be opposite. Applied chemometric approach to data analysis confirmed our claim that the direction of lipid profile modifications induced by PSO varies in physiological and pathological conditions and in case of many FAs and CFAs it appears to be opposite. It also confirmed our previous claim that dietary supplements may exert opposite effect, depending from the condition of the organism. That is why unauthorized use of dietary supplements by patients suffering from different disorders may be counterproductive and may result in health deterioration. In our opinion the biological activity of dietary supplements should be extensively examined before their admission for use to ensure their safety for patients.

## Figures and Tables

**Table 1:** Fatty acids content in hepatic microsomes of experimental groups (µg/g of fat).

Fatty Acid	Group								*p* Value
	CON	M	G	GM	CONplus	Mplus	Gplus	GMplus	
C12:0	3.22 ± 2.03	4.11 ± 2.60	4.26 ± 2.45	4.94 ± 1.63	4.77 ± 2.65	4.34 ± 1.49	5.22 ± 3.16	4.49 ± 1.66	ns
C14:0	13.5 ± 10.8 ^a,b,c^	17.7 ± 12.4 ^d,e^	19.6 ± 5.96 ^f^	18.3 ± 5.83 ^g^	28.8 ± 6.61 ^a^	22.9 ± 9.19	40.1 ± 23.2 ^b,d^	38.9 ± 9.02 ^c,e,f,g^	<0.0001
C15:0	13.4 ± 9.36 ^a,b^	13.5 ± 8.96 ^c,d^	11.1 ± 1.74 ^e,f^	13.7 ± 1.74 ^g^	12.9 ± 4.15 ^h,i^	17.4 ± 6.90	29.1 ± 9.46 ^a,c,e,h^	33.7 ± 11.3 ^b,d,f,g,i^	<0.0001
C16:0 (mg/g)	1.22 ± 0.67	1.40 ± 0.90	1.12 ± 0.11	1.38 ± 0.23	1.10 ± 0.34	1.78 ± 0.67	1.75 ± 0.64	1.49 ± 0.43	ns
C17:0	57.9 ± 37.6 ^a^	64.4 ± 48.5 ^b^	45.3 ± 8.22 ^c,d,e^	56.9 ± 9.25	64.8 ± 23.5	102 ± 37.7 ^c^	95.9 ± 29.5 ^d^	107 ± 35.2 ^a,b,e^	<0.0001
C18:0 (mg/g)	1.73 ± 0.90	2.06 ± 1.51	1.60 ± 0.24 ^a^	1.92 ± 0.27	1.76 ± 0.54	2.71 ± 0.93 ^a^	1.89 ± 0.55	2.02 ± 0.54	0.0209
C20:0	4.26 ± 2.97	6.64 ± 4.50	4.24 ± 1.50	4.91 ± 1.46	2.86 ± 1.12 ^a^	3.64 ± 1.23	4.29 ± 2.30	6.04 ± 2.88 ^a^	0.0198
C21:0	3.55 ± 2.67	3.42 ± 2.11	2.87 ± 1.65	3.51 ± 2.17	2.46 ± 1.12	2.02 ± 0.15	5.52 ± 3.47	4.47 ± 2.00	ns
C22:0	4.56 ± 1.96	1.64 ± 0.81	2.32 ± 0.20	2.75 ± 2.12	nd	nd	5.10 ± 2.76	3.85 ± 2.63	ns
Sum of SFA (mg/g)	3.04 ± 1.63	3.57 ± 2.47	2.80 ± 0.34 ^a^	3.40 ± 0.49	2.97 ± 0.90	4.63 ± 1.58 ^a^	3.82 ± 1.09	3.57 ± 0.86	0.0051
c7C16:1	11.4 ± 7.87 ^a^	13.8 ±12.1 ^b^	11.0 ± 5.27 ^c^	12.1 ± 4.82	15.9 ± 5.10	12.0 ± 4.47	23.8 ± 9.62 ^a,b,c^	15.6 ± 4.20	0.0031
c9C16:1	42.9 ± 29.5 ^a,b^	59.1 ± 46.6	45.0 ± 16.5 ^c,d^	50.7 ± 19.2	76.9 ± 39.2	77.4 ± 35.8	110 ± 54.7 ^a,c^	101 ± 40.0 ^b,d^	0.0001
c9C17:1	7.17 ± 5.14 ^a,b^	6.67 ± 4.44 ^c,d^	5.39 ± 2.55 ^e,f^	6.71 ± 2.77 ^g,h^	10.0 ± 5.75	10.6 ± 4.98	23.5 ± 11.7 ^a,c,e,g^	19.8 ± 3.75 ^b,d,f,h^	<0.0001
t9C18:1	3.77 ± 3.32	4.87 ± 3.12	4.27 ± 2.25	5.33 ± 1.72	3.24 ± 0.97	5.39 ± 3.33	5.10 ± 3.59	6.44 ± 2.09	ns
c6C18:1	6.61 ± 5.99	5.81 ± 4.94	3.78 ± 1.23	6.43 ± 2.50 ^a^	2.31 ± 1.11 ^a,b^	5.53 ± 2.67 ^b^	4.59 ± 2.50	3.63 ± 2.22	0.0031
c9C18:1	529 ± 306	646 ± 413	436 ± 134	512 ± 130	477 ± 135	597 ± 184	749 ± 371	566 ± 172	ns
c11C18:1	92.2 ± 57.8 ^a^	104 ± 68.9 ^b^	71.8 ± 14.2 ^c,d,e,f^	89.7 ± 13.8 ^g^	142 ± 55.8 ^c^	228 ± 85.6 ^a,b,d,g^	179 ± 71.9 ^e^	163 ± 40.3 ^f^	<0.0001
c14C18:1	nd	nd	nd	nd	1.66 ± 0.00	3.16 ± 1.55	4.12 ± 2.07	7.07 ± 3.89	ns
c11C20:1	3.78 ± 2.61	4.78 ± 4.06	4.73 ± 2.11	4.48 ± 2.20	5.84 ± 2.09	5.40 ± 2.32	8.11 ± 4.35	7.30 ± 2.38	0.0281
c5C24:1	11.3 ± 6.41	11.3 ± 7.03	7.76 ± 3.31	8.62 ± 5.42	14.9 ± 13.2	nd	18.7 ± 2.07	12.6 ± 4.74	ns
Sum of MUFA (mg/g)	0.70 ± 0.42	0.85 ± 0.55	0.58 ± 0.15^a^	0.69 ± 0.17	0.74 ± 0.23	0.90 ± 0.32	1.10 ± 0.45^a^	0.83 ± 0.31	0.0192
c9c12C18:2 (mg/g)	1.08 ± 0.62	1.21 ± 0.77	1.03 ± 0.13	1.10 ± 0.17 ^a^	0.61 ± 0.24 ^a,b^	1.27 ± 0.58 ^b^	0.96 ± 0.42	0.96 ± 0.45	0.0135
c6c9c12C18:3	8.25 ± 3.97 ^a,b^	12.9 ± 9.35	19.8 ± 6.13 ^a,c^	13.2 ± 4.90	9.77 ± 5.91 ^c,d^	25.3 ± 12.7 ^b,d^	11.9 ± 8.63	10.4 ± 3.73	<0.0001
c11c14C20:2	5.19 ± 3.10 ^a,b^	6.93 ± 4.77 ^c,d^	5.32 ± 2.65 ^e,f^	7.00 ± 1.87 ^g^	8.15 ± 3.35	9.62 ± 4.29	13.8 ± 4.56 ^a,c,e^	14.9 ± 4.47 ^b,d,f,g^	<0.0001
c8c11c14C20:3	23.3 ± 12.2	31.6 ± 27.6	21.2 ± 6.12 ^a,b^	30.8 ± 4.42	24.4 ± 9.40	29.5 ± 12.0	50.7 ± 30.6 ^a^	46.9 ± 22.5 ^b^	0.0003
c5c8c11c14C20:4 (mg/g)	1.20 ± 0.66 ^a^	1.59 ± 1.24 ^b^	1.37 ± 0.22 ^c^	1.53 ± 0.23	1.53 ± 0.54	2.73 ± 1.06 ^a,b,c^	1.52 ± 0.47	1.68 ± 0.54	0.0013
c9c12c15C18:3	70.0 ± 50.3 ^a,b^	71.3 ± 41.5 ^c,d^	70.4 ± 21.4 ^e,f,g,h^	73.5 ± 16.9 ^i,j,k,l^	10.4 ± 4.27 ^a,c,e,i^	13.6 ± 6.00 ^b,d,f,j^	18.8 ± 7.61 ^g,k^	21.5 ± 9.22 ^h,l^	<0.0001
c5c8c11c14c17C20:5	37.2 ± 17.6 ^a,b^	51.2 ± 37.5 ^c,d^	44.8 ± 16.8 ^e,f^	46.6 ± 9.4 ^g,h^	12.2 ± 8.9 ^a,c,e,g^	10.6 ± 4.5 ^b,d,f,h^	29.1 ± 19.1	23.8 ± 10.2	<0.0001
c7c10c13c16c19C22:5	77.4 ± 40.8	83.8 ± 59.2	60.8 ± 9.31	86.1 ± 9.68 ^a^	37.0 ± 17.4 ^a,b,c^	71.0 ± 39.8	96.5 ± 53.7 ^b^	105.1 ± 56.6 ^c^	0.0002
c4c7c10c13c16c19C22:6	518 ± 300 ^a^	579 ± 405 ^b^	469 ± 56.1^c^	591 ± 113	608 ± 236	1072 ± 413 ^a,b,c^	601 ± 156	664 ± 211	0.0008
c9t11C18:2 CLA	nd	nd	15.7 ± 3.36	23.1 ± 7.76 ^a,b^	nd	nd	7.05 ± 3.63 ^a^	5.35 ± 4.58 ^b^	<0.0001
Sum of PUFA (mg/g)	3.01 ± 1.69 ^a^	3.63 ± 2.57 ^b^	3.09 ± 0.39	3.40 ± 0.43	2.85 ± 1.03	5.23 ± 2.01 ^a,b^	3.30 ± 0.97	3.53 ± 1.28	0.0124
Sum of n3 PUFA	703 ± 401	785 ± 537 ^a^	638 ± 71.5 ^b^	791 ± 113	668 ± 257	1166 ± 455 ^a,b^	744 ± 208	815 ± 270	0.0083
Sum of n6 PUFA	2311 ± 1290 ^a^	2842 ± 2045 ^b^	2440 ± 330	2589 ± 382	2184 ± 777 ^c^	4060 ± 1561 ^a,b,c^	2549 ± 775	2711 ± 1007	0.0142
n6/n3	3.31 ± 0.31 ^a^	3.62 ± 0.56	3.82 ± 0.30 ^a,b,c^	3.43 ± 0.27	3.32 ± 0.23 ^b^	3.49 ± 0.23	3.43 ± 0.41	3.29 ± 0.27 ^c^	0.0095

Data are presented as mean values ± standard deviation. Values marked with the same letter index in the row were significantly different in Dunn’s test (*p* ≤ 0.05). nd—not detected. ns—not significant. c—*cis*; t—*trans*; CLA—conjugated linoleic acid; SFA—saturated fatty acids; MUFA—monounsaturated fatty acids; PUFA—polyunsaturated fatty acids; n3 PUFA—n3 polyunsaturated fatty acids; n6 PUFA—n6 polyunsaturated fatty acids.

**Table 2:** Contents of geometrical CFA isomers in hepatic microsomes of experimental groups (µg/g of fat).

	Group								*p* Value
	CON	M	G	GM	CONplus	Mplus	Gplus	GMplus	
CFA	42.0 ± 25.0 ^a,b,c^	41.1 ± 15.8 ^d,e,f^	343 ± 136 ^a,d,g,h^	371 ± 173 ^b,e,i,j^	29.8 ± 7.48 ^g,i,k,l^	36.0 ± 11.6 ^h,j,m,n^	264 ± 229 ^k,m^	690 ± 469 ^c,f,l,n^	<0.0001
CD	31.8 ± 19.3 ^a,b,c,d^	34.6 ± 18.1 ^e,f,g,h^	304 ± 118 ^a,e,i,j^	325 ± 149 ^b,f,k,l^	28.7 ± 6.66 ^i,k,m,n^	38.4 ± 15.6 ^j,l,o^	247 ± 209 ^c,g,m^	443 ± 194 ^d,h,n,o^	<0.0001
tt	18.8 ± 10.0 ^a,b,c,d^	23.1 ± 13.9 ^e,f,g^	82.1 ± 35.6 ^a,e,h,i^	85.8 ± 36.4 ^b,f,j,k^	16.9 ± 4.34 ^h,j,l,m^	22.7 ± 8.50 ^i,k,n^	93.7 ± 77.0 ^c,l^	210 ± 136 ^d,g,m,n^	<0.0001
ct	7.77 ± 6.23 ^a,b,c^	10.1 ± 6.63 ^d,e,f^	216 ± 83.3 ^a,d,g,h^	232 ± 112 ^b,e,i,j^	5.59 ± 1.79 ^g,i,k,l^	10.5 ± 5.82 ^h,j,m^	94.7 ± 78.4 ^k^	308 ± 283 ^c,f,l,m^	<0.0001
c9t11CLA	7.57 ± 5.27 ^a,b,c^	8.02 ± 6.40 ^d,e,f^	199 ± 78.1 ^a,d,g,h^	217 ± 108 ^b,e,i,j^	3.68 ± 1.16 ^g,i,k,l^	8.87 ± 6.26 ^h,j,m^	62.5 (18.4–212) ^k^	247 ± 238 ^c,f,m^	<0.0001
cc	3.22 ± 2.67 ^a,b^	3.43 ± 1.95^c^	6.24 ± 2.24 ^d^	7.17 ± 4.12 ^e^	6.83 ± 2.31	5.19 ± 2.11 ^f^	12.1 (5.27–27.6) ^a^	49.5 ± 28.8 ^b,c,d,e,f^	<0.0001
CT	3.31 (1.34–8.15) ^a,b^	2.97 (1.07–8.26) ^c,d^	38.8 ± 24.3 ^a,c,e,f^	45.4 ± 28.6 ^b,d,g,h^	0.59 ± 0.57 ^e,g,i^	0.98 ± 0.67 ^f,h,j^	4.68 (1.10–19.9)	20.9 (5.53–78.8) ^i,j^	<0.0001
*ttt*	5.20 (0.77–35.2) ^a^	6.42 (0.89–46.5)	33.71 ± 24.56	40.67 ± 22.75 ^a^	nd	nd	23.8 (0.88–645)	157.75 ± 0.00	ns
ttc/ctt	1.71 ± 1.12	2.28 ± 1.55	3.82 ± 2.75 ^a^	1.33 (0.60–2.98) ^b^	0.39 ± 0.27 ^a,c,d^	0.81 ± 0.67 ^e^	7.78 ± 5.64 ^c^	15.6 ± 12.6 ^b,d,e^	<0.0001
cct	1.74 ± 1.50 ^a^	2.00 (0.64–6.31)	3.85 ± 1.97	6.89 ± 3.60 ^b^	1.91 ± 0.00	0.50 ± 0.27 ^b,c^	3.74 (0.53–26.2)	158 (36.1–699) ^a,c^	0.0007

Data are shown as mean values ± standard deviation (SD). Variables with skew distribution were transformed into logarithms, retransformed after calculations and presented as mean and confidence interval. *p* value ≤ 0.05—significant differences among groups according to Kruskal—Wallis test. Values sharing a letter are statistically different according to Dunn’s test. CD—conjugated dienes, CFA—conjugated fatty acids, CLA—conjugated linoleic acid, CT—conjugated trienes, c—*cis*, t—*trans*, nd—not detected, ns—not significant.

**Table 3:** Profiles of geometrical CFA isomers in hepatic microsomes of experimental groups.

		Group								*p* Value
		CON	M	G	GM	CONplus	Mplus	Gplus	GMplus	
CD (%CFA)		80.7 ± 23.2 ^a,b^	85.1 ± 18.6	89.2 ± 3.7 ^c,d^	88.6 ± 4.3 ^e,f^	96.6 ± 4.6 ^a,c,e^	97.8 ± 2.0 ^b,d,f^	94.9 ± 5.2	87.3 ± 26.7	<0.0001
	tt (%CD)	63.3 ± 16.2 ^a,b^	64.7 ± 11.3 ^c,d^	26.7 ± 3.5 ^a,c,e,f^	27.1 ± 4.3 ^b,d,g,h^	59.2 ± 9.2 ^e,g^	59.9 ± 4.1 ^f,h^	41.9 ± 15.5	41.2 ± 11.6	<0.0001
	ct (%CD)	27.5 ± 14.3 ^a,b^	31.5 ± 12.9 ^c,d^	71.1 ± 3.5 ^a,c,e,f^	70.6 ± 4.1 ^b,d,g,h^	20.1 ± 6.4 ^e,g,i^	26.4 ± 4.3 ^f,h^	40.0 ± 22.2	52.0 ± 14.8 ^i^	<0.0001
	cc (%CD)	9.3 ± 5.2	9.2 ± 2.2	2.2 ± 0.8 ^a,b,c^	2.3 ± 1.0 ^d,e^	22.8 ± 5.7 ^a,d^	13.8 ± 3.2 ^b,e^	10.2 ± 5.9 ^c^	9.7 ± 4.3	<0.0001
	c9t11CLA (%CFA)	23.0 ± 9.6 ^a,b^	20.3 ± 12.1 ^c,d^	58.4 ± 4.9 ^a,c,e,f^	58.2 ± 4.0 ^b,d,g,h^	12.9 ± 4.6 ^e,g,i^	20.2 ± 4.5 ^f,h^	38.3 ± 15.4 ^i^	37.4 ± 16.7	<0.0001
	c9t11CLA (%CD)	26.5 ± 10.7 ^a,b^	25.2 ± 12.5 ^c,d^	65.4 ± 3.9 ^a,c,e,f^	65.7 ± 3.9 ^b,d,g,h^	13.4 ± 4.8 ^e,g,I,j^	21.9 ± 6.1 ^f,h^	40.8 ± 17.2 ^i^	42.4 ± 15.8 ^j^	<0.0001
CT (%CFA)		13.4 ± 11.0 ^a,b^	7.0 (2.5–25.1)	10.8 ± 3.7 ^c,d^	11.4 ± 4.3 ^e,f^	2.2 ± 2.2 ^a,c,e^	2.6 ± 1.9 ^b,d,f^	4.6 ± 4.2	6.7 ± 6.0	<0.0001
	ttt (%CT)	49.2 ± 24.8	36.7 ± 3.7	80.4 ± 19.0	75.8 ± 26.8	-	-	-	-	n.s.
	ttc/ctt (%CT)	51.7 ± 35.4	61.1 ± 30.5 ^a^	11.7 ± 8.5 ^b,c^	11.8 ± 29.3 ^a,d,e,f^	90.7 ± 29.5 ^b,d^	61.6 ± 30.1	77.7 ± 35.6 ^c,e^	72.5 ± 42.8 ^f^	<0.0001
	cct (%CT)	35.9 ± 21.2	53.7 ± 22.7	21.3 ± 17.3	17.0 ± 13.0 ^a^	93.3 ± 2.5	66.3 ± 34.3 ^a^	49.0 ± 38.1	82.6 ± 8.7	0.0084

Data are shown as mean values ± standard deviation (SD). Variables with skew distribution were transformed into logarithms, retransformed after calculations and presented as mean and confidence interval. *p* value ≤ 0.05—significant differences among groups according to Kruskal–Wallis test. Values sharing a letter are statistically different (*p* ≤ 0.05) according to Dunn’s test. CD—conjugated dienes, CFA—conjugated fatty acids, CLA—conjugated linoleic acid, CT—conjugated trienes, c—*cis*, t—*trans*, nd—not detected, ns—not significant.

**Table 4:** Coefficients and average value of canonical variables included in the final model.

Coefficients of Canonical Variables		
Variable (Discriminatory Power)	DF1 (88.5%)	DF2 (11.5%)
c9c11C18:2	2.80842	−0.30746
c14C18:1	−2.18297	1.43115
c7C15:1	0.10547	−0.95926
c15C24:1	−0.89805	−0.15973
ttc/ctt	−1.25729	−0.25606
CT	1.30678	0.53155
c6C18:1	0.23964	0.56732
ct	0.38138	0.77035
c6c9c12C18:3	0.48747	−0.34854
cc	−0.06846	−0.54010
C15:0	−0.41474	0.71331
c9C17:1	0.62505	−0.03485
c11C20:1	−0.38061	−0.02071
t9C18:1	−0.35859	−0.27043
c7c10c13c16c19C22:5	−0.33071	−0.46821
C20:0	0.09110	0.28954
c11c14C20:2	0.43479	0.08553
Average Value of Canonical Variables		
Sk1	6.80004	−0.12432
Sk2	−1.94173	2.35397
Sk3	−2.42916	−1.11482

**Table 5:** Classification results of LDA presenting percentage predicted clusters membership for actual clusters.

Actual Cluster	Correct Classification (%)	Predicted Cluster Classification		
		Sk1	Sk2	Sk3
Sk1	100.0	22	0	0
Sk2	81.8	0	18	4
Sk3	97.7	1	0	43
All	94.3	23	18	47

**Table 6:** Peroxidability index, LA isomerization index and indices of the desaturase activity in hepatic microsomes.

	Group								*p* Value
	CON	M	G	GM	CONplus	Mplus	Gplus	GMplus	
PI	117 ± 4.63 ^a^	118 ± 11.7 ^b^	120 ± 21.2	125 ± 6.41	128 ± 10.9 ^c^	141 ± 7.47 ^a,b,d,e^	113 ± 11.8 ^c,d^	125 ± 18.0 ^e^	<0.0001
D4D	6.74 ± 1.06 ^a,b^	6.91 ± 0.80 ^c,d^	7.80 ± 0.97	6.84 ± 0.70 ^e,f^	17.2 ± 3.86 ^a,c,e^	16.6 ± 3.66 ^b,d,f^	7.38 ± 2.98	7.33 ± 2.83	<0.0001
D5D	52.7 ± 10.4 ^a^	52.3 ± 11.9 ^b^	68.6 ± 18.8 ^c,d^	50.7 ± 11.7 ^e^	64.9 ± 18.7 ^f^	95.3 ± 18.5 ^a,b,e,g,h^	36.1 ± 13.9 ^c,f,g^	39.8 ± 12.1 ^d,h^	<0.0001
D9D_C16	0.03 ± 0.01 ^a,b,c^	0.04 ± 0.02	0.04 ± 0.01	0.04 ± 0.01 ^d,e,f^	0.07 ± 0.04 ^a,d^	0.04 ± 0.01	0.07 ± 0.03 ^b,e^	0.07 ± 0.02 ^c,f^	<0.0001
D9D_C18	0.31 ± 0.06 ^a^	0.35 ± 0.19	0.25 ± 0.05	0.27 ± 0.07	0.29 ± 0.09	0.21 ± 0.08 ^a,b^	0.42 ± 0.24 ^a,b^	0.29 ± 0.06	0.0010
D9D_total	0.19 ± 0.03	0.22 ± 0.10	0.17 ± 0.03	0.17 ± 0.04	0.20 ± 0.06	0.14 ± 0.04 ^a^	0.23 ± 0.07 ^a^	0.18 ± 0.06	0.0390
Iso-LA	-	-	0.02 ± 0.00	0.02 ± 0.01 ^a^	-	-	0.01 ± 0.00	0.01 ± 0.01 ^a^	<0.0001

Data are shown as mean values ± standard deviation (SD). P value ≤ 0.05—significant differences among groups according to Kruskal–Wallis test. Values sharing a letter are statistically different according to Dunn’s test. D4D—Δ-4 desaturase index, D5D—Δ-5 desaturase index, D9D_C16—Δ-9 desaturase index for C16 FA, D9D_C18—Δ-9 desaturase index for C18 FA, D9D_total—total Δ-9 desaturase index, Iso-LA—LA isomerization index, PI—peroxidability index.

## Data Availability

Data available on request. The data presented in this study are available on request from the corresponding author. The data are not publicly available due to privacy restrictions of the collaborators.
